# Metabolic Response to Daytime Dry Fasting in Bahá’í Volunteers—Results of a Preliminary Study

**DOI:** 10.3390/nu14010148

**Published:** 2021-12-29

**Authors:** Anja Mähler, Carmen Jahn, Lars Klug, Caroline Klatte, Andreas Michalsen, Daniela Koppold-Liebscher, Michael Boschmann

**Affiliations:** 1 Experimental and Clinical Research Center (ECRC), a Cooperation between Charité—Universitätsmedizin Berlin and Max-Delbrück-Center for Molecular Medicine, 13125 Berlin, Germany; carmen.jahn@posteo.de (C.J.); larsklug@yahoo.de (L.K.); michael.boschmann@charite.de (M.B.); 2 Berlin Institute of Health, Corporate Member of Freie Universität Berlin and Humboldt Universität zu Berlin, Charité—Universitätsmedizin Berlin, 10117 Berlin, Germany; 3 Max-Delbrück-Center for Molecular Medicine (MDC) in the Helmholtz Association, 13125 Berlin, Germany; 4 DZHK (German Centre for Cardiovascular Research), 10785 Berlin, Germany; 5 Institute of Social Medicine, Epidemiology & Health Economics, Corporate Member of Freie Universität Berlin and Humboldt-Universität zu Berlin, Charité—Universitätsmedizin Berlin, Charitéplatz 1, 10117 Berlin, Germany; caroline.klatte@charite.de (C.K.); a.michalsen@immanuel.de (A.M.); d.liebscher@immanuel.de (D.K.-L.); 6 Department of Internal and Integrative Medicine, Immanuel Krankenhaus Berlin, 14109 Berlin, Germany

**Keywords:** religious fasting, daytime dry fasting, energy expenditure, body composition, microdialysis

## Abstract

Each year in March, adherents of the Bahá’í faith abstain from eating and drinking from sunrise to sunset for 19 days. Thus, Bahá’í fasting (BF) can be considered as a form of daytime dry fasting. We investigated whether BF decreased energy expenditure after a meal and whether it improved anthropometric measures and systemic and tissue-level metabolic parameters. This was a self-controlled cohort study with 11 healthy men. We measured anthropometric parameters, metabolic markers in venous blood and pre- and postprandial energy metabolism at systemic (indirect calorimetry) and tissue (adipose tissue and skeletal muscle microdialysis) level, both before and during BF. During BF, we found reduced body weight, body mass index, body fat and blood glucose. Postprandial increase in energy expenditure was lower and diet-induced thermogenesis tended to be lower as well. In adipose tissue, perfusion, glucose supply and lipolysis were increased. In skeletal muscle, tissue perfusion did not change. Glucose supply and lipolysis were decreased. Glucose oxidation was increased, indicating improved insulin sensitivity. BF may be a promising approach to losing weight and improving metabolism and health. However, outside the context of religiously motivated fasting, skipping a meal in the evening (dinner cancelling) might be recommended, as metabolism appeared to be reduced in the evening.

## 1. Introduction

Various fasting regimens are becoming increasingly popular in Western societies where constant, easy access to energy-dense, palatable foods prevails. Concomitantly, deleterious health consequences, such as obesity, cardiovascular disease, diabetes and cancer, necessitate preventative, as well as complementary, treatment options. One promising fasting regimen that promotes cardiometabolic health seems to be the modulation of meal timing [1].

A long-known and frequently researched practice of mealtime modulation is Ramadan fasting. There are a number of studies on the metabolic effects of Ramadan fasting in healthy individuals. A meta-analysis of 30 cohort studies showed that Ramadan fasting decreases low-density lipoprotein and fasting glucose levels in both men and women. Men additionally lost weight [2]. A study that tried to elucidate the cause of this weight loss failed to show changes in resting and total energy expenditure and concluded that weight loss was probably due to changes in food intake [3]. This was in line with an earlier study that also found unchanged resting and total energy expenditure in Ramadan fasters [4]. Alsubheen et al. used another approach to investigate energy expenditure in this context. They measured substrate oxidation in the morning and in the evening and found that it shifted from carbohydrate to lipid oxidation [5]. This shift was likely caused by the depletion of glycogen stores during the daytime fasting period.

Another form of religiously motivated daytime dry fasting is Bahá’í fasting (BF). The Bahá’í religion was founded in Iran in 1863 and has been growing ever since. Nowadays, it has followers all over the world [6]. Every year before the beginning of the Bahá’í New Year on March 19/20, Bahá’ís perform a nineteen-day fast in which they abstain from eating and drinking from sunrise to sunset. Although this fast resembles Ramadan fasting, it has one unique feature. Whereas Ramadan is a lunar month for which daylight hours vary from year to year, BF is always practiced in March when day and night are nearly of equal duration (equinox). This unique feature prompted us to investigate the metabolic response to 12 h daytime dry fasting in healthy adherents of the Bahá’í faith.

Since studies on Ramadan fasting did not show changes in total and resting energy expenditure, we designed our study to investigate the thermic effect of food, i.e., diet-induced thermogenesis, which accounts for approximately 10% of total energy expenditure. Moreover, there is still controversy on whether early- or late-time-restricted eating is more favorable for health, although some evidence points towards an advantage of eating in the morning and early afternoon [7]. This prompted us to investigate diet-induced thermogenesis following the same meal in the morning vs. in the evening in the context of BF.

We recently reported circadian clock changes in healthy Bahá’í men and women during their BF [8]. Here, we report using a subset of this cohort, in which we tested if BF decreased energy expenditure after a meal and improved anthropometric, systemic and tissue-level metabolic parameters.

This research is not only relevant for adherents, but also for Western populations who tend to consume large proportions of food in the evening. A study, which used a smartphone app, revealed that daily eating patterns in healthy adults are highly variable and eating is only discontinued during sleep. Fifty percent of adults eat for more than 15 h every day. Restricting eating to 10–11 h daily decreased weight and improved sleep quality [9].

## 2. Materials and Methods

### 2.1. Study Design

This was a sub-study of a large prospective exploratory cohort study with 144 participants, which investigated the medical and psychological effects of BF (ClinicalTrials.gov identifier: NCT03443739). The sub-study was carried out at the Experimental and Clinical Research Center of Charité—Universitätsmedizin Berlin, from February to March 2018. Data on the primary and other secondary endpoints were published elsewhere [8,10].

### 2.2. Participants

Participants were screened at the Department of Integrative Medicine of the Institute of Social Medicine, Epidemiology and Health Economics and then referred to this sub-study. Enrollment took place between January and February 2018. We included members of the Bahá’í religious community, aged between 18 and 69 years, who intended to perform the BF. Key exclusion criteria were body mass index below 18.0 and above 30.9 kg/m^2^, planned interruption of BF of more than 5 days and severe physical and psychological illnesses or known eating disorders. Bahá’ís only enter their yearly fast if they are currently healthy and they are allowed to discontinue the fast if a health condition occurs.

This study was conducted in accordance with the 1964 Declaration of Helsinki and its following amendments. The institutional review board of Charité-niversitätsmedizin Berlin approved the study protocol (EA4/216/17), and all participants gave written informed consent before they entered the study.

### 2.3. Intervention

All participants abstained from eating, drinking and smoking from sunrise to sunset from 1–19 March 2018 as part of their religious practice.

### 2.4. Outcome Measures

Outcome measures were predetermined and did not change during or after the study. The primary outcome measure of this sub-study was diet-induced thermogenesis after a meal, assessed by indirect calorimetry before vs. during BF. Secondary outcome measures were body mass index, body fat percentage, and systemic and tissue-level glucose and lipid metabolism before vs. during BF.

### 2.5. Sample Size Calculation

This sub-study was an exploratory study aimed at generating hypotheses for future confirmatory studies. Therefore, no previous sample size calculation was made. The chosen number of participants was based on methodological and feasibility considerations.

### 2.6. Clinical Protocols

**Figure 1:**
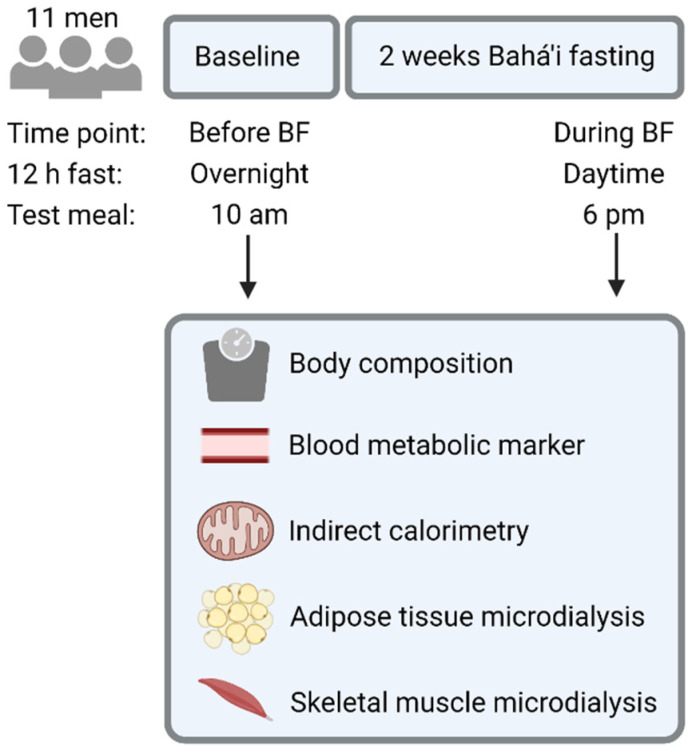
Study design of the self-controlled cohort study on Bahá’i fasting (BF). Figure created with BioRender.com (accessed on 27 December 2021).

All participants were assessed the week before and in the last week of their fast (Figure 1). Therefore, assessments before BF and during BF started in the morning after a 12 h overnight fast and in the evening after a 12 h daytime fast, respectively. We asked participants to abstain from caffeine, alcohol and vigorous exercise for 24 h before study protocols.

The study protocol started with the determination of each participant’s body composition by air displacement plethysmography (Bod Pod, Life Measurements, Inc., Concord, CA, USA). All other measurements were performed in the supine position after a 30 min resting period. Blood was drawn from a catheter in a large antecubital vein at regular intervals. We employed indirect calorimetry to measure carbon dioxide (CO_2_) production and oxygen (O_2_) consumption (Quark RMR, COSMED, Rome, Italy). From this, we calculated changes in energy expenditure and the respiratory exchange ratio (RER = VCO_2_/VO_2_).

In addition, we performed microdialysis of abdominal subcutaneous adipose tissue and the vastus lateralis muscle in a subset of five participants. Microdialysis is a minimally invasive technique for continuous, in-vivo monitoring of easily accessible tissues. It provides insights into interstitial concentration changes of marker metabolites for glucose and lipid metabolism. Procedures were as described elsewhere [11], except that M71 microdialysis probes (µDialysis, Stockholm, Sweden) were used.

After baseline measurements, participants received a meal (bread, butter, curd, cheese and cucumber) prepared by a dietician, which provided 441 kcal with 50, 30 and 20% of energy from carbohydrates, fats and proteins, respectively. This was at 10 am before BF and at 6 pm during BF. Afterwards, we continued calorimetry for 4 h with 15 min breaks at the beginning of each hour. Blood and dialysis samples were taken at regular intervals until the end of testing.

### 2.7. Assays

Plasma glucose, insulin and triglyceride concentrations were measured according to international standards. The microdialysis dialysate/perfusate ethanol ratio served as a marker for changes in tissue perfusion [12]; glucose, lactate and pyruvate served as marker metabolites for glycolysis; and glycerol served as a marker metabolite for lipolysis. The ethanol ratio, as well as dialysate glucose, lactate, pyruvate and glycerol concentrations, were measured as described previously [11].

### 2.8. Data Analysis

This was an exploratory study. Therefore, we performed no sample size calculation or adjustments for multiple testing. Statistical analyses were performed with GraphPad Prism (version 8.0.0). To determine if data were normally distributed, we checked distributions visually by using histograms, boxplots and Q–Q plots. Furthermore, we evaluated the difference between means and medians and rated the skewness.

Owing to the normal distribution, data were presented as mean and standard deviation (SD) in tables and as mean and standard error of mean (SEM) in figures. Differences (before vs. during BF) were compared by paired student’s *t*-test. Two-way ANOVA was used for multiple comparisons and *p* values for fasting effects (before vs. during BF) are shown in the figures. A *p* value < 0.05 indicated statistical significance.

## 3. Results

Table 1 shows the baseline characteristics of 11 male participants. Mean body mass index indicated that subjects were slightly overweight. All participants completed the BF as planned and there were no dropouts.

### 3.1. Anthropometry

There were small but significant reductions in body weight (1.7 ± 1.0 kg, *p* = 0.0002), body mass index (0.5 ± 0.3 kg/m^2^, *p* = 0.0003) and body fat (1.1 ± 1.6%, *p* = 0.04) during vs. before BF. Lean mass did not change due to fasting (before BF: 60.7 ± 5.6 kg, during BF: 60.3 ± 6.0 kg).

### 3.2. Systemic Metabolism

**Figure 2:**
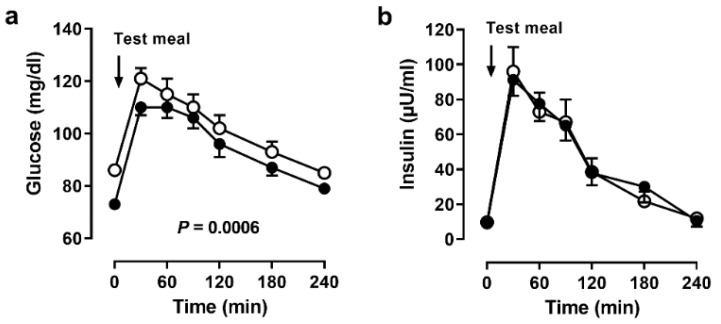
Systemic responses to the meal. Serum (**a**) glucose and (**b**) insulin concentrations before and after a meal in 11 men before (open circles) and during (closed circles) Bahá’í fasting. Data are presented as means (SEM), *p* values by ANOVA.

Plasma glucose concentrations were lower during BF, both before and after the meal (Figure 2a). This decrease was not accompanied by a change in serum insulin concentrations (Figure 2b). There were no significant changes of fasting triglycerides (before BF: 88 ± 43 mg/dL, during BF: 80 ± 39 mg/dL).

**Figure 3:**
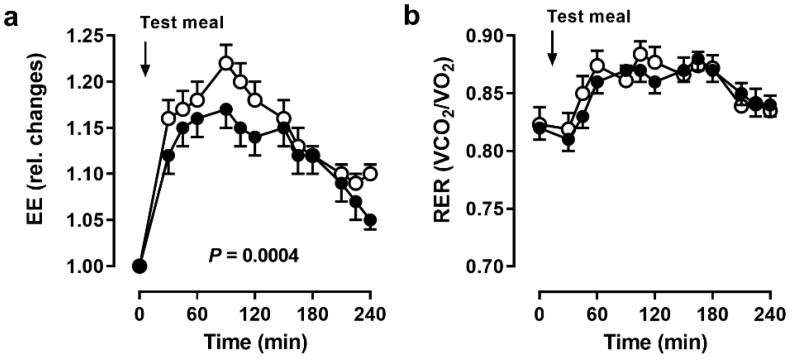
Systemic energy metabolism. (**a**) Energy expenditure (EE) and (**b**) respiratory exchange ratio (RER) before and after a meal in 11 men before (open circles) and during (closed circles) Bahá’í fasting. Data are presented as mean (SEM), *p* values by ANOVA.

Resting energy expenditure did not change due to fasting (before BF: 1746 ± 176 kcal/d, during BF: 1726 ± 224 kcal/d). However, the increase in energy expenditure after the meal was markedly blunted during BF (*p* = 0.0004, Figure 3a). Notably, energy expenditure did not reach baseline values until the end of testing.

Resting RER remained virtually the same (before BF: 0.82 ± 0.05, during BF: 0.82 ± 0.04). After a short decrease in RER after the meal, it increased for almost 2 h and then decreased until the end of testing without reaching baseline values (Figure 3b).

### 3.3. Primary Outcome Measure

Diet-induced thermogenesis following the 441 kcal meal tended to be lower during BF (before BF: 9.8 ± 2.3%, during BF: 7.9 ± 3.0%, *p* = 0.1).

### 3.4. Adipose Tissue Metabolism

**Figure 4:**
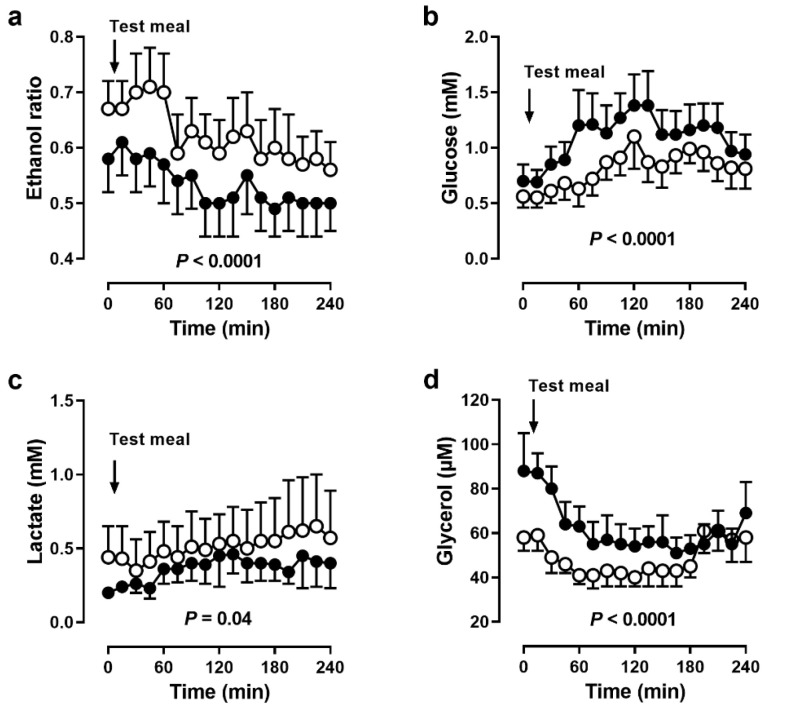
Adipose tissue microdialysis. (**a**) Ethanol ratio and dialysate concentrations of (**b**) glucose, (**c**) lactate and (**d**) glycerol in adipose tissue before and after a meal in 5 men before (open circles) and during (closed circles) Bahá’í fasting. Data are presented as means (SEM), *p* values by ANOVA.

In adipose tissue, ethanol ratios were lower during BF, indicating higher tissue perfusion (*p* < 0.0001, Figure 4a). After the meal, ethanol ratios slightly decreased both before and during BF. Accordingly, glucose concentrations were higher (*p* < 0.0001, Figure 4b) and lactate concentrations were lower during BF (*p* = 0.04, Figure 4c). Glycerol concentrations were markedly higher during BF (*p* < 0.0001, Figure 4d).

### 3.5. Skeletal Muscle Metabolism

**Figure 5:**
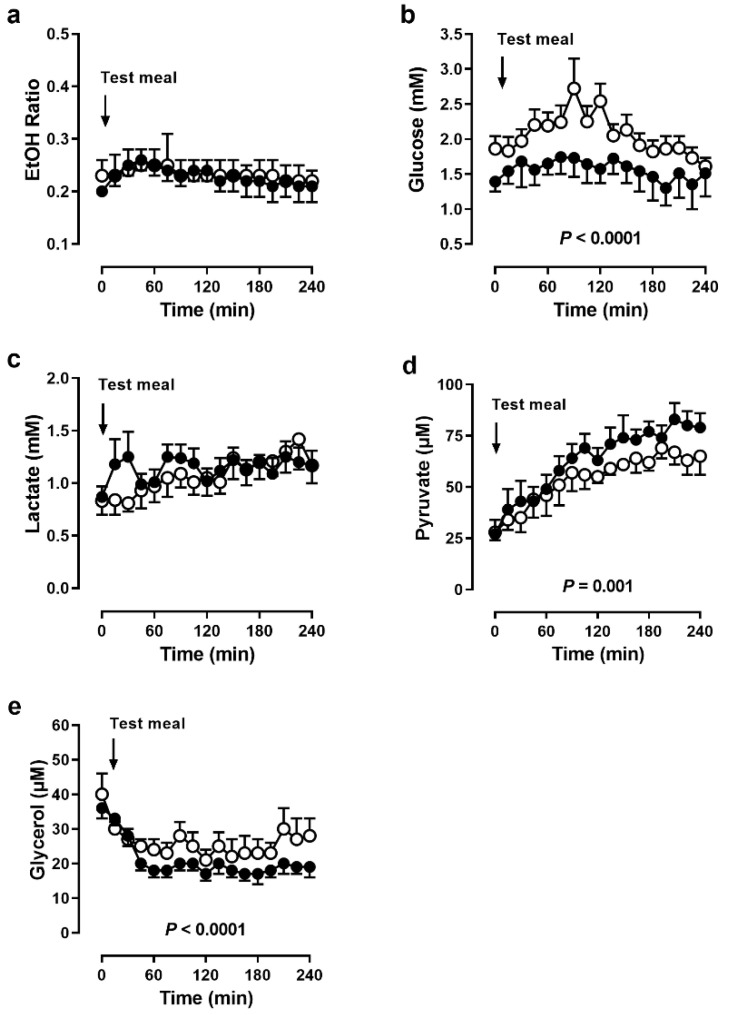
Skeletal muscle microdialysis. (**a**) Ethanol ratio and dialysate concentrations of (**b**) glucose, (**c**) lactate, (**d**) pyruvate and (**e**) glycerol in skeletal muscle before and after a meal in 5 men before (open circles) and during (closed circles) Bahá’í fasting. Data are presented as means (SEM), *p* values by ANOVA.

In skeletal muscle, ethanol ratios were virtually the same before and during BF (Figure 5a). Despite unchanged tissue perfusion, glucose concentrations were lower during BF (*p* < 0.0001, Figure 5b). Lactate concentrations did not change due to fasting (Figure 5c), but pyruvate concentrations were higher during BF (*p* = 0.001, Figure 5d). Glycerol concentrations were lower during BF, especially in the postprandial phase (*p* = 0.001, Figure 5e).

## 4. Discussion

Different types of intermittent fasting have become increasingly popular in Western populations. Here, we tested whether BF decreased energy expenditure after a meal and improved anthropometric, systemic and tissue-level metabolic parameters. Our study design allowed us to investigate effects of short-term dry fasting and meal timing in BF.

Participants experienced slight reductions in body weight (2%). This was in line with a meta-analysis of 30 studies in healthy, male Ramadan fasters [2]. Body fat decreased while lean mass remained unchanged. These reductions suggest a fasting effect. This might result from reduced meal frequency and/or reduced calorie intake. A weight loss regimen that reduces body weight but maintains lean mass is not only appealing for adherents but also for people who want to practice a type of intermittent fasting.

We found lower glucose concentrations during BF. Since measurements during BF were performed in the evening, lower blood glucose was probably a time-of-day effect. Although fasting time was the same as before BF, participants were more active during the day, which would have depleted their glycogen stores faster. Insulin concentrations did not change due to fasting. Interestingly, even though participants consumed a rather moderate 441 kcal breakfast, insulin concentrations needed 4 h to reach almost basal levels. Consequently, meals should be at least 4 h apart to avoid constantly increased insulin levels.

In line with three Ramadan studies [3,4,5], resting energy expenditure did not change due to BF. This was to be expected, since body weight reduction was not clinically relevant. However, postprandial energy expenditure measured over 4 h was lower during BF. With a reduction of 20%, diet-induced thermogenesis tended to be lower. The thermogenic response to food is not only determined by energy content and nutrient composition, but also by circadian variation with lowest values in the evening [13]. Lower values indicate that less of the ingested calories are expanded in metabolic processes but stored in the body. This finding is relevant for increasingly overweight and obese societies. In addition, breakfast skipping increased the risk of mortality from circulatory diseases and all causes in Japanese men and women [14], and atherosclerosis risk in a Spanish cohort [15]. Healthy subjects that consumed their daily energy requirement in one evening meal for 8 weeks, exhibited glucose intolerance in the morning [16]. Furthermore, a recent study showed that diet-induced thermogenesis was 2.5 times higher in normal-weight men after a high-calorie breakfast vs. a high-calorie dinner [17]. Taken together, it seems advisable to eat the greater proportion of daily calories in the morning.

RER and, therefore, substrate utilization did not change due to fasting. Alsubheen et al. found differences in substrate utilization in Ramadan fasters when comparing morning and evening measurements within the fasting period [5]. However, since our participants had fasted for 12 h both before and during BF, they were in a comparable metabolic state. 

This was the first study that used the microdialysis technique to assess metabolic changes locally in adipose tissue and skeletal muscle during fasting. In adipose tissue, the ethanol ratio was lower and, therefore, tissue perfusion higher during BF. This could have been caused by decreased body fat, since adipose tissue perfusion depends on the degree of obesity [18]. Decreased body fat and increased tissue perfusion suggested a fasting effect of BF. Changes in tissue perfusion affected substrate supply and removal of metabolic products and thus dialysate metabolite concentrations. In line with this, dialysate glucose and lactate concentrations were higher and lower during BF, respectively. Furthermore, dialysate glycerol concentrations were also higher during BF. Taking into account an increased tissue perfusion, and therefore product removal, actual tissue lactate and glycerol could have been even higher than indicated by dialysate concentrations.

Higher adipose tissue lactate and glycerol concentrations are indicators for an increased lipolytic state. At their arrival in the evening, participants were in a highly lipolytic state (1.5-fold higher basal dialysate glycerol concentrations). This difference was also most likely caused by the difference between a 12 h overnight and a 12 h daytime fast. Whole-body lipolysis increases early in fasting, within 14 h after the last meal [19]. Our results supported this finding on a cellular level in adipose tissue.

The skeletal muscle constitutes the major postprandial glucose disposal route. In skeletal muscle, tissue perfusion remained unchanged. Therefore, the differences in metabolite concentrations were not attributable to tissue perfusion. However, dialysate glucose concentrations were lower during BF. This could be related to the lower blood glucose concentrations, because absolute dialysate glucose concentrations are identical with blood glucose concentrations [20]. However, the much lower postprandial increase in dialysate glucose concentrations indicated a rather increased glucose uptake in skeletal muscle. Postprandial dialysate lactate concentrations did not change, indicating that anaerobic glycolysis remained unchanged. However, skeletal muscle pyruvate concentrations were increased, specifically in the late postprandial phase. Therefore, the higher postprandial glucose uptake during BF resulted primarily in an improved aerobic glycolysis. The increases in glucose uptake and oxidative metabolism were indicative of improved insulin sensitivity.

This study had a number of limitations. It was conducted in a small group of 11 healthy men, which limited the generalization of our findings to women, children, elderly and underweight individuals. The small sample size resulted from the fact that the fasting interval was generally fixed for the time March 1–19. In order to sample baseline data as close as possible to the beginning of fasting, we studied 1–2 men each at days −6, −5, −4, −3, −2 and −1 before BF. For the same reasons, follow-up data were collected at days 14–19 during BF. We did not think that this shifted data collection influenced the metabolic outcome of BF, since metabolic adaptations occurred normally during the early days of fasting. Additionally, due to the comprehensive study protocol, we could not test more than two volunteers per day. There was no control group, so we cannot exclude the effect of time on outcomes. Due to the religious nature of BF, it was impossible to perform the testing during BF in the morning. Therefore, it was not always fully clear if outcomes were due to fasting or time-of-day effects. It would have been interesting to evaluate possible changes of calorie intake or physical activity, because their relation to the metabolic outcomes cannot be excluded. However, this study could serve as a draft in order to design more sophisticated and controlled clinical trials on BF in the future.

## 5. Conclusions

Based on our results, BF, as a form of daytime dry fasting, can be considered physiologically harmless over a period of two weeks. Further studies with more than one fasting period might provide evidence on health promoting effects of BF. Skipping a meal in the evening (dinner cancelling) might be preferred, as metabolism appears to be reduced in the evening.

## Figures and Tables

**Table 1:** Baseline characteristics of healthy men before Bahá’í fasting ^1^.

Characteristic	
*n*	11
Age, years	38 (14)
Body mass index, kg/m^2^	26 (4)
Body fat, %	25 (12)
Waist circumference, cm	95 (15)
Fasting glucose, mg/dL	86 (7)
Fasting insulin, µU/mL	9.7 (5.6)
HOMA-IR	2.1 (1.3)
Fasting triglycerides, mg/dL	88 (43)

^1^ Data are presented as means (SD). HOMA-IR, homeostasis model assessment of insulin resistance.

## Data Availability

Data described in this manuscript will be made available upon request.

## References

[1] Swiatkiewicz I., Wozniak A., Taub P.R. (2021). Time-Restricted Eating and Metabolic Syndrome: Current Status and Future Perspectives. Nutrients.

[2] Kul S., Savas E., Ozturk Z.A., Karadag G. (2014). Does Ramadan fasting alter body weight and blood lipids and fasting blood glucose in a healthy population? A meta-analysis. J. Relig. Health.

[3] Lessan N., Saadane I., Alkaf B., Hambly C., Buckley A.J., Finer N., Speakman J.R., Barakat M.T. (2018). The effects of Ramadan fasting on activity and energy expenditure. Am. J. Clin. Nutr..

[4] McNeil J., Mamlouk M.M., Duval K., Schwartz A., Nardo Junior N., Doucet E. (2014). Alterations in metabolic profile occur in normal-weight and obese men during the Ramadan fast despite no changes in anthropometry. J. Obes..

[5] Alsubheen S.A., Ismail M., Baker A., Blair J., Adebayo A., Kelly L., Chandurkar V., Cheema S., Joanisse D.R., Basset F.A. (2017). The effects of diurnal Ramadan fasting on energy expenditure and substrate oxidation in healthy men. Br. J. Nutr..

[6] Demmrich S. (2020). How to Measure Baha’i Religiosity: The CRSi-20 for Baha’is as a First Reliable and Valid Measurement. Religions.

[7] Charlot A., Hutt F., Sabatier E., Zoll J. (2021). Beneficial Effects of Early Time-Restricted Feeding on Metabolic Diseases: Importance of Aligning Food Habits with the Circadian Clock. Nutrients.

[8] Koppold-Liebscher D.A., Klatte C., Demmrich S., Schwarz J., Kandil F.I., Steckhan N., Ring R., Kessler C.S., Jeitler M., Koller B. (2021). Effects of Daytime Dry Fasting on Hydration, Glucose Metabolism and Circadian Phase: A Prospective Exploratory Cohort Study in Baha’i Volunteers. Front. Nutr..

[9] Gill S., Panda S. (2015). A Smartphone App Reveals Erratic Diurnal Eating Patterns in Humans that Can Be Modulated for Health Benefits. Cell Metab..

[10] Demmrich S., Koppold-Liebscher D., Klatte C., Steckhan N., Ring R.M. (2021). Effects of religious intermittent dry fasting on religious experience and mindfulness: A longitudinal study among Baha’is. Psychol. Relig. Spiritual..

[11] Mahler A., Steiniger J., Bock M., Klug L., Parreidt N., Lorenz M., Zimmermann B.F., Krannich A., Paul F., Boschmann M. (2015). Metabolic response to epigallocatechin-3-gallate in relapsing-remitting multiple sclerosis: A randomized clinical trial. Am. J. Clin. Nutr..

[12] Fellander G., Linde B., Bolinder J. (1996). Evaluation of the microdialysis ethanol technique for monitoring of subcutaneous adipose tissue blood flow in humans. Int. J. Obes. Relat. Metab. Disord..

[13] Romon M., Edme J.L., Boulenguez C., Lescroart J.L., Frimat P. (1993). Circadian variation of diet-induced thermogenesis. Am. J. Clin. Nutr..

[14] Yokoyama Y., Onishi K., Hosoda T., Amano H., Otani S., Kurozawa Y., Tamakoshi A. (2016). Skipping Breakfast and Risk of Mortality from Cancer, Circulatory Diseases and All Causes: Findings from the Japan Collaborative Cohort Study. Yonago Acta Med..

[15] Uzhova I., Fuster V., Fernandez-Ortiz A., Ordovas J.M., Sanz J., Fernandez-Friera L., Lopez-Melgar B., Mendiguren J.M., Ibanez B., Bueno H. (2017). The Importance of Breakfast in Atherosclerosis Disease: Insights from the PESA Study. J. Am. Coll. Cardiol..

[16] Carlson O., Martin B., Stote K.S., Golden E., Maudsley S., Najjar S.S., Ferrucci L., Ingram D.K., Longo D.L., Rumpler W.V. (2007). Impact of reduced meal frequency without caloric restriction on glucose regulation in healthy, normal-weight middle-aged men and women. Metabolism.

[17] Richter J., Herzog N., Janka S., Baumann T., Kistenmacher A., Oltmanns K.M. (2020). Twice as High Diet-Induced Thermogenesis After Breakfast vs Dinner on High-Calorie as Well as Low-Calorie Meals. J. Clin. Endocrinol. Metab..

[18] Adams F., Jordan J., Schaller K., Luft F.C., Boschmann M. (2005). Blood flow in subcutaneous adipose tissue depends on skin-fold thickness. Horm. Metab. Res..

[19] Soeters M.R., Soeters P.B., Schooneman M.G., Houten S.M., Romijn J.A. (2012). Adaptive reciprocity of lipid and glucose metabolism in human short-term starvation. Am. J. Physiol. Endocrinol. Metab..

[20] Bolinder J., Ungerstedt U., Arner P. (1992). Microdialysis measurement of the absolute glucose concentration in subcutaneous adipose tissue allowing glucose monitoring in diabetic patients. Diabetologia.

